# The critical role of ferritinophagy in human disease

**DOI:** 10.3389/fphar.2022.933732

**Published:** 2022-09-08

**Authors:** Meng-Zhen Liu, Ni Kong, Guang-Yu Zhang, Qin Xu, Yang Xu, Ping Ke, Chong Liu

**Affiliations:** Department of Pharmacy, Second Military Medical University, Shanghai, China

**Keywords:** ferroptosis, ferritinophagy, iron homeostasis, NCOA4, heme degradation

## Abstract

Ferritinophagy is a type of autophagy mediated by nuclear receptor activator 4 (NCOA4), which plays a role in inducing ferroptosis by regulating iron homeostasis and producing reactive oxygen species in cells. Under physiological conditions, ferritinophagy maintains the stability of intracellular iron by regulating the release of free iron. Studies have demonstrated that ferritinophagy is necessary to induce ferroptosis; however, under pathological conditions, excessive ferritinophagy results in the release of free iron in large quantities, which leads to lipid peroxidation and iron-dependent cell death, known as ferroptosis. Ferritinophagy has become an area of interest in recent years. We here in review the mechanism of ferritinophagy and its association with ferroptosis and various diseases to provide a reference for future clinical and scientific studies.

## Introduction

Ferroptosis is an iron-dependent, non-apoptotic mechanism of cell death, and the production of reactive oxygen species (ROS) and lipid peroxidation leading to plasma membrane damage are the core events leading to ferroptosis. The main marker of ferroptosis is phospholipid hydroperoxides (PLOOHs), which is composed of phospholipids containing polyunsaturated fatty acids (PUFAs) in cell membranes (PUFA-PLs) (Jiang et al., 2021). Lipidomics studies have shown that phosphatidylethanolamine is more strongly associated with ferroptosis than other phospholipid types. Long-chain acyl-CoA synthetase 4 (ACSL4) can catalyze the esterification of arachidonic acid and adrenal acid into phosphatidylethanolamine (Jiang et al., 2021). Besides, ACSL4 binds free PUFAs to coenzyme A (CoA) to produce PUFA-CoAs, which then binds to PLs and blocks ferroptosis. Studies have shown that the inhibition of system Xc^−^ uptake can inhibit the synthesis of glutathione (GSH) and glutathione peroxidase 4 (GPX4), leading to peroxide accumulation and ferroptosis (Stockwell et al., 2017). GPX4 catalyzes the generation of non-toxic and low-reactivity lipid alcohols from PLOOHs to inhibit ROS production, which plays an important role in inhibiting ferroptosis (Ursini and Maiorino, 2020). Some lipid oxygenases (LOXs) are non-heme iron-dependent dioxygenases that directly oxidize PUFAs of biofilms, suggesting that LOXs may mediate the induction process of ferroptosis. Heme oxygenase 1 (HO-1)–mediated degradation of heme releases iron, which is closely related to ferroptosis (Dong et al., 2020; Ma et al., 2020).

Iron is a central cofactor for several proteins involved in constitutive cell functions. Both iron deficiency and iron overload negatively affect physiology (Bulvik et al., 2012). When the level of bioavailable iron is low, the body regulates the release of iron and supplemental ferritin through selective autophagy degradation (Lawson et al., 1991). When autophagy is activated, NCOA4 mediates the binding of ferritin to the lysosome, causing free iron to be released by degrading ferritin. This type of selective autophagy is known as “ferritinophagy” (Mancias et al., 2014). NCOA4 promotes the autophagic degradation of ferritin through direct binding, which results in the formation of ferritinophagy. In contrast, the inhibition of autophagy leads to the accumulation of NCOA4, which further confirms that NCOA4 is the autophagic receptor of ferritin (Mancias et al., 2015). Therefore, the proper maintenance of NCOA4 levels in cells is important for the regulation of ferritinophagy. Ferritinophagy causes increased intracellular iron levels and triggers the accumulation of ROS through the Fenton reaction, resulting in ferroptosis (Gao et al., 2016). This cell death mode is closely related to the occurrence and development of many diseases (Stockwell et al., 2017). Dysregulation of iron metabolism and ferroptosis have been observed in neurodegeneration, cancer, and infection; however, little is known regarding the role of ferritinophagy in the pathogenesis of these diseases. NCOA4-mediated ferritinophagy has been implicated in the maintenance of efficient erythropoiesis through iron release for mitochondrial heme synthesis. Recently, ferritinophagy has become an active area of research.

This study reviews the underlying mechanism of ferritinophagy and expounds the relationship between ferritinophagy and ferroptosis. The relationship between ferritinophagy and disease development is also discussed to provide a theoretical basis for future clinical and scientific research.

## Regulatory mechanism of ferritinophagy

**FIGURE 1:**
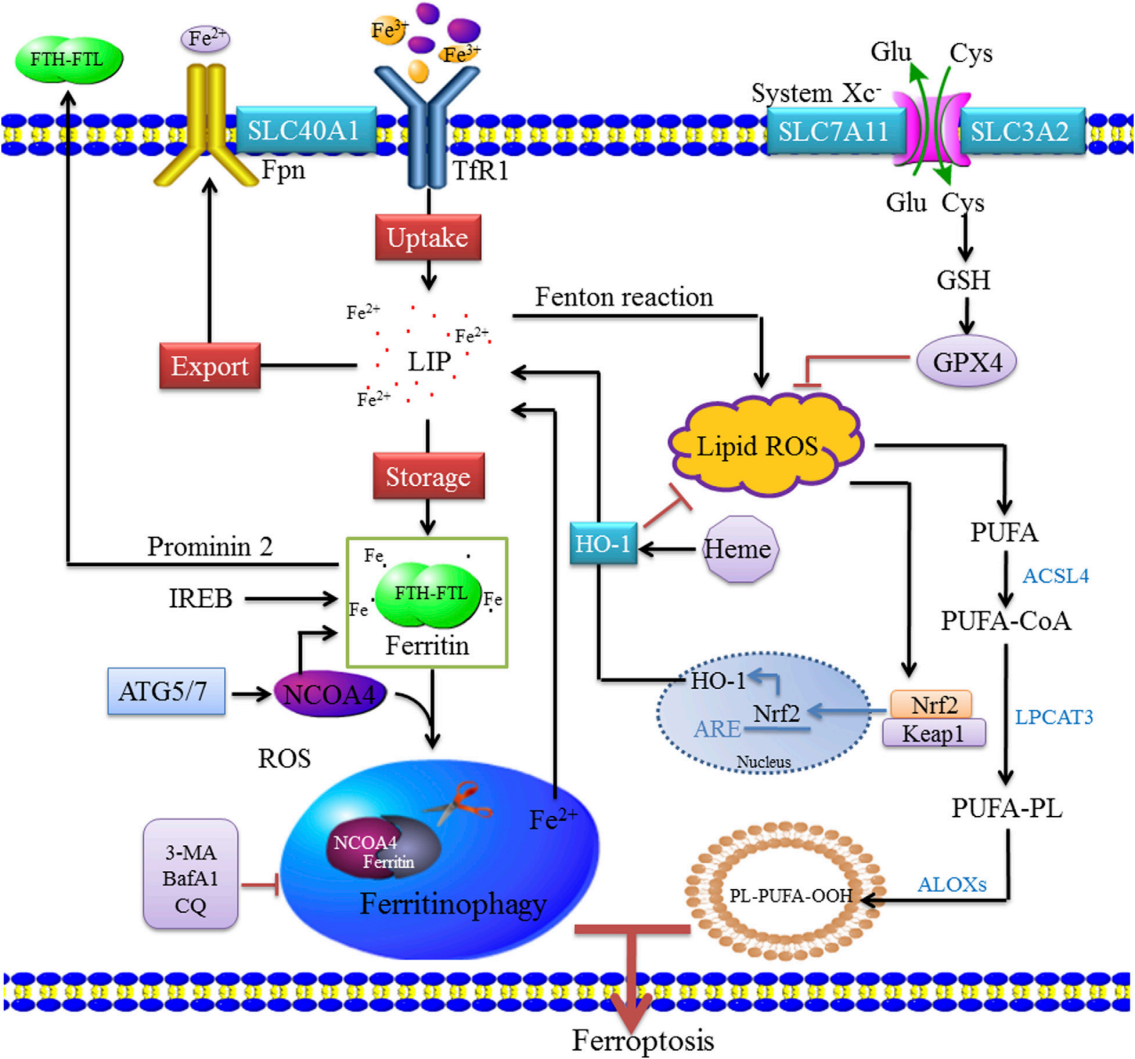
Molecular mechanisms of ferritinophagy. Extracellular Fe^3+^ relies on Tfr1 for transferring into the cell to form Fe^2+^, where it binds to form a ferritin storage cage, which is composed of FTL and FTH. Fe^3+^ is reduced to Fe^2+^ in the endosome by STEAP3 (iron reductase), which then mediates the release of Fe^2+^ from the endosome into the cytoplasm via DMT1/SLC11A2 to form LIP. Cytoplasmic Fe^2+^ can be reduced by ferritin and stored as Fe^3+^, and Fe^2+^ can be transported out of the cell by ferroportin (FPN1/SLC40A1). These mechanisms of controlling iron intake, storage, and export maintain the amount of iron in the redox-active LIP. When bioavailable iron levels are low, the body regulates the release of iron and replenishes ferritin through selective autophagy degradation. When autophagy is activated, NCOA4 mediates the binding of ferritin to the lysosome, causing it to degrade and release free iron. NCOA4 promotes its autophagic degradation by binding ferritin, leading to the formation of ferritinophagy, whereas the inhibition of autophagy leads to the accumulation of NCOA4. Ferritinophagy leads to increased intracellular iron levels and ROS accumulation through the Fenton reaction, leading to ferroptosis. ACSL4 is involved in the biosynthesis of PUFAs and helps free PUFAs to be esterified and incorporated into membrane phospholipids. Nrf2 activates HO-1, which leads to an increase in heme degradation. An imbalance among these homeostatic factors could lead to an accumulation of excess intracellular iron, increased ROS production, and induction of cell death.

The multicellular process modifies the sensitivity of cells to ferroptosis by altering the intracellular unstable iron content. Iron uptake, storage, and export are strictly regulated, and the regulation of iron homeostasis provides a complete ferroptosis network. Increased iron uptake, decreased iron export, or decreased iron storage all can lead to iron overload and promote the occurrence and development of ferroptosis, and various iron chelating agents can inhibit ferroptosis (Sumneang et al., 2020; Yamada et al., 2020). At present, the factors regulating intracellular iron concentration mainly include the following: 1) iron uptake—extracellular Fe^3+^ binds to transferrin (Tf) and is located in endosomes by transferrin receptor (TfR1)–mediated endocytosis (Song et al., 2021). In the slightly acidic endosomal environment, Fe^3+^ is separated from Tf and reduced to Fe^2+^ by the metal reductase STEAP3 with the increase in pH. After reduction to Fe^2+^, non-transferrin-bound iron was directly transported to cell via divalent metal ion transporter 1 (DMT1) (Doll and Conrad, 2017). Heme iron is absorbed directly into cells by binding to heme carrier protein 1 (HCP1). In addition, the upregulation of nuclear factor erythroid 2–related factor 2 (Nrf2) activates HO-1, which leads to increased heme entry into the cytoplasm, heme degradation, and release of free iron from mitochondria (Fang et al., 2019); 2) iron export—The part of Fe^2+^ in cytoplasm is stored in the form of ferritin, and the other part enters the circulation through ferroportin (Fpn/SLC40A1). After iron enters the circulation, it is oxidized by hephaestin or ceruloplasmin to Fe^3+^, which binds to transferrin. Additionally, ferritin can be transported by the Prominin-2 polyvesicle–exosome pathway out of the cell when the cell is overloaded with iron (Gao et al., 2016; Zhou et al., 2018; Feng H et al., 2020); and 3) iron storage—Fe^2+^ enters the cells to form cytoplasmic labile iron pool (LIP) (Kakhlon and Cabantchik, 2002; Feng J et al., 2020). One part of Fe^2+^ is stored as ferritin, and the other part is used for various biochemical processes. Heme degradation and NCOA4-mediated autophagy can increase the instability of the LIP; intracellular accumulation of Fe^2+^ can be triggered by the Fenton reaction (Van der Riet et al., 1985; Gammella et al., 2016; Chen et al., 2020). HO-1 is a rate-limiting enzyme in the process of heme degradation (Ma et al., 2022), which catalyzes the oxidative degradation of heme and the release of free iron, carbon monoxide (CO), and biliverdin (Facchinetti, 2020). The activation of HO-1 leads to increased heme degradation and Fe^2+^-mediated ferritin expression, resulting in an increase in iron levels in LIP (Wang et al., 2019). In the process of hemoglobin anabolism, Nrf2 regulates ferroptosis by regulating heme mobilization of iron during heme catabolism, promoting iron storage, reducing iron accumulation, and upregulating SLC7A11 activity to increase glutamate content (Liu et al., 2021). Nrf2 is regulated by P62, which inhibits Nrf2 degradation and transcriptional function and downregulates SLC7A11 expression, thereby effectively inhibiting ferroptosis. HO-1 is highly expressed in organs responsible for the degradation of aged red blood cells, and HO-1 in macrophages is involved in the circulation of heme (Choi and Kim, 2022). However, the overexpression of HO-1 promotes cancer cell proliferation and survival (Drummond et al., 2019). In addition, HO-1 induces angiogenesis by regulating the expression of angiogenic factors (Huang et al., 2021). Other studies have shown that HO-1 knockout mice develop anemia associated with iron overload in the liver and kidney, resulting in oxidative tissue damage and chronic inflammation (Li H. B et al., 2020; Yang et al., 2021). These findings indicate that HO-1 is not only involved in normal physiology but also has a role in pathophysiological states, and plays a dual regulatory role in ferroptosis (Chiang et al., 2018) (Figure 1).

Ferritinophagy is a selective form of autophagy, and excessive ferritinophagy can induce ferroptosis (Mancias et al., 2014). Ferritin is the primary iron storage protein in the body, and its content and activity affect cellular iron levels. However, cells lacking NCOA4 cannot degrade ferritin, which results in a decrease in intracellular bioavailable iron. This suggests that NCOA4 is a selective cargo receptor for ferritin autophagy conversion, which is essential for iron homeostasis. NCOA4 is activated by the autophagy-related gene (ATG), which guides ferritin heavy chain (FTH) and ferritin light chain (FTL) to the autophagosome. The knockout of NCOA4 or ATG (ATG3, ATG5, ATG7, etc.) can significantly decrease ferritinophagy, iron overload, lipid peroxidation, and ferroptosis (Hou et al., 2016). In addition, intracellular iron levels were regulated by ferrimodulin 1 (IRP1) and ferrimodulin 2 (IRP2) post-transcriptional levels (Rouault, 2006; Bayeva et al., 2013). The consumption of NCOA4 increases the level of IRP2, an RNA-binding protein that binds to a set of irreversible electroporation to regulate MMA. When iron content is sufficient, IRP1 contains Fe-S clusters and IRP2 degrades, thus inhibiting the IRP system (Fang et al., 2022). When the IRP system is unable to maintain adequate levels of intracellular iron during severe iron deficiency, the iron preservation pathway is activated and involved in maintaining intracellular iron balance. In addition, NCOA4 mRNA is induced during erythropoiesis and its expression is associated with genes related to heme biosynthesis (Griffiths et al., 2012). Traditionally, NCOA4-mediated ferritinophagy occurs in the cytoplasm; however, ferritin may also be degraded to induce iron poisoning and ferroptosis in the mitochondria. It is unknown whether or not this process depends on NCOA4 and the underlying mechanism of ferritin degradation in the mitochondria. Cytoplasmic free iron enters mitochondria through mitochondrial ferritin (MFRN), which is used for iron–sulfur clusters and heme biosynthesis (Campanella et al., 2009). Excess iron can be stored in a unique form in mitochondrial ferritin (FTMT) (Wang et al., 2016). An increase in FTMT in hypoxic human macrophages is associated with decreased NCOA4 expression. NCOA4 expression may be reduced by impaired transcriptional regulation during hypoxia, and its mRNA is degraded by microRNA 6862-5p, whereas c-jun N-terminal kinase is involved in regulating the degradation of microRNA 6862-5p (Fuhrmann et al., 2020). Hara et al. (2020) reported that the iron loss induces MFRN through the hypoxia-inducible factor 1α (HIF-1α)–specific protein 1 axis and triggers mitophagy for damaged mitochondria. Ferritinophagy maintains iron stability *in vivo* by regulating the release of free iron. When iron levels are low, the transferrin activity is increased, ferritinophagy is activated, and intracellular iron content increases in various ways. When iron levels are high, HERC2 binds to the C-terminus of NCOA4 through its Cullin 7 (CUL7) homology domain. This results in a decrease in NCOA4 in a ubiquitin-dependent manner, which inhibits ferritinophagy and blocks the release of iron from ferritin (Moroishi et al., 2014).

In addition to ferritinophagy, other types of autophagy are associated with ferroptosis, such as lipophagy, clockophagy, and chaperone-mediated autophagy (CMA). Lipophagy is the process by which lipid droplets (LDs) in cells are selectively transported by the autophagosome for lysosome degradation. RAB7A is a cargo protein that mediates the degradation of LDs and plays an important role in regulating the autophagy lysosome. Enhancing tumor protein D52 (TPD52)–dependent lipid storage or blocking ATG5/RAB7A-dependent lipid degradation prevents RSL3-induced lipid peroxidation and subsequent ferroptosis *in vitro* and *in vivo* (Bai et al., 2019). The overexpression of TPD52 promotes lipid storage and alleviates ferroptosis caused by RSL3. “Clockophagy,” the selective degradation of the core circadian clock protein, ARNTL, by autophagy, is necessary for ferroptosis. SQSTM1 acts as a cargo receptor for the degradation of autophagic ARNTL, which inhibits ferroptosis by repressing Egln2 transcription, thus activating the pro-survival transcription factor, HIF-1α. Therefore, the activation of the autophagy-dependent SQSTM1-ARNTL-EGLN2-HIF-1α pathway mediates lipid peroxidation and promotes ferroptosis (Yang et al., 2019). CMA is a cellular lysosome-mediated degradative mechanism. HSP90 inhibits GPX4 degradation and promotes ferroptosis by regulating the stability of lysosomal surface proteins during CMA (Wu et al., 2019). LAMP2A and HSPA8/HSC70 regulate CMA, which leads to erastin-induced GPX4 degradation in HT22 cells. In addition to selective autophagy, autophagy regulators (e.g., SLC7A11, BECN1, STAT3, Nrf2, p53, HSPB1, and ACSL4) also contribute to ferroptosis; however, the precise role of autophagy regulatory factors in ferroptosis remains unknown.

## Ferritinophagy and related disease

As our understanding of ferroptosis/ferritinophagy increases, more studies have revealed that ferritinophagy-mediated ferroptosis is involved in the occurrence and development of neurological, infectious, cardiovascular, and cerebrovascular diseases and tumors. In contrast, the diseases induced by ferroptosis have a negative impact on human health. On the contrary, ferroptosis also represents a new anticancer strategy.

### Ferritinophagy and neurodegenerative diseases

Iron plays an important role in DNA synthesis, mitochondrial respiration, and neurotransmitter synthesis, and changes in iron homeostasis in patients with neurodegenerative diseases (ND) are associated with decreased autophagy. Defects in the autophagy lysosomal pathway may be associated with the pathogenesis of ND, oxidative stress induces lysosome rupture in ferroptosis, whereas phagocytic vesicles formed during autophagy are bound to lysosomes and degraded. Currently, activating or inhibiting ferroptosis may be exploited to achieve therapeutic effects in ND (Tang et al., 2018).

The death of dopaminergic neurons in the substantia nigra during PD is associated with the accumulation of iron (Jiang et al., 2017; Zucca et al., 2017; Song and Xie, 2018). PD is characterized by the depletion of dopamine neurons in the substantia nigra of the brain and abnormal aggregation of Lewy bodies, in which α-synuclein (α-syn) is regulated by iron during translation (Febbraro et al., 2012). Iron directly interacts with α-syn in Lewy bodies, and α-syn is mainly digested through an autophagy lysosomal pathway, which not only responds to iron chelators and channels but also participates in the cellular iron cycle through the degradation of ferritin and other iron-containing components. These studies suggest that there is a relationship between iron deposition and autophagy injury during PD (Chen L. L et al., 2019). Baksi and Singh (2017) demonstrated that α-syn affects ferritinophagy by disrupting lysosome activity, thus leading to PD. This suggests that ferritinophagy may be involved in the progression of PD. Several PD causative or associated mutations involve proteins whose functions are associated with autophagy. PD-associated mutations in genes encoding parkin, PTEN-induced putative kinase 1, Parkinson protein 7, and HtrA serine peptidase 2 result in deficits in mitochondrial homeostasis and mitophagy pathway. RNA sequencing (RNA-seq) revealed that FTH1 was abnormally expressed in 6-hydroxydopamine (6-OHDA)–induced rat PD. In PC-12 cells, FTH1 overexpression downregulates NCOA4, inhibits ferritinophagy and ferroptosis, and ameliorates cell death. The autophagy inhibitors, chloroquine and bafilomycin A (1), can significantly inhibit ferritinophagy and ferroptosis in PC-12 cells treated with 6-OHDA (Tian et al., 2020). Since redox-active iron is associated with the generation of ROS, inappropriate iron accumulation may be a key factor in the pathogenesis of PD.

HD (**Huntington's disease**) is an autosomal dominant ND characterized by a loss of motor control, loss of neurons in the striatum and cortex, and cognitive decline that leads to dementia. Both autophagy and CMA are either activated or impaired in patients with HD and animal models (McColgan and Tabrizi, 2018). Koga observed an increased CMA activity in different cell and mouse models of HD.

AD is characterized by abnormal concentrations of β-amyloid and tau proteins inside and outside cells, which leads to neuronal dysfunction and neurodegeneration. Recent studies have shown that iron deposited in brain tissue is a pathogenic factor of AD and induces oxidative stress that leads to the death of nerve cells (Mena et al., 2015). However, the relationship between iron homeostasis and the pathogenesis of AD remains unclear. Proteins involved in the pathogenesis of AD may contribute to the autophagy pathway or iron homeostasis. The autophagy lysosomal pathway appears to be significantly involved in the pathogenesis of AD, playing a pivotal role in the production and accumulation of amyloid plaques and neurofibrillary tangles. Furthermore, the stimulation of the autophagic clearance of protein aggregates is a potential therapeutic strategy to restore behavioral, learning, and memory deficits and limit AD progression (Biasiotto et al., 2016).

With the reduction in global autophagy observed in some ND, ferritin autophagy flow is also reduced. *In vitro* models of NCOA4 depletion indicate that bioavailable iron decreases as a result of reduced ferritin degradation, which leads to impaired iron release (Bellelli et al., 2016). Studies on the role of autophagy in iron processing provide a new perspective on the role of iron in neurodegeneration, suggesting that autophagy dysregulation may lead to the dysregulation of iron homeostasis. Overall, further studies are needed to understand the role of NCOA4 in central nervous system development and the pathophysiology of iron-related ND.

### Ferritinophagy and urinary tract infections

Ferroptosis induced by autophagy is an important regulatory pathway associated with vascular endothelial damage caused by inflammatory inducers (Flores-Mireles et al., 2015). Urinary tract infections (UTIs) are generally caused by uropathogenic *Escherichia coli* (UPEC) that persist in the urinary tract epithelial cells. Ferritin-bound iron to UPEC shuttles between the autophagosome and lysosomal compartments in the urinary tract epithelium. Iron is very important for the survival of host cells. Increasing the content of free iron in cells significantly increases the growth rate of the host cells. Therefore, a high dependence of UPEC on iron may be used as a novel strategy to defend against pathogens (Khasheii et al., 2016).

Bauckman and Mysorekar (2016) first reported that NCOA4-dependent ferritinophagy triggers excessive proliferation and persistence of UPEC in host cells, which is caused by increased intracellular iron availability. Iron overload in urinary epithelial cells induces ferritinophagy in an NCOA4-dependent manner. This results in increased iron availability of UPEC, leading to excessive bacterial proliferation and host cell death. In addition, the lysosome damage caused by iron overload represents a specific mechanism that causes host cell death.

Ferritin phagocytosis promotes erythropoiesis and the development of UTIs as follows: 1) During iron deficiency, NCOA4 selectively interacts with the FTH1 subunit of ferritin through its C-terminal domain and arginine residues on FTH1. NCOA4 binds to PCBP1 and mediates ferritin flux, which is crucial for regulating heme synthesis and further erythrocyte differentiation. Concurrently, NCOA4-mediated ferritinophagy accelerates UTIs by increasing the availability of iron, thus enabling the persistence of UPEC in host cells. 2) In the case of adequate iron, HERC2 mediates the degradation of NCOA4 in a ubiquitin-dependent manner through the CUL7 homologous domain of HERC2 and the C-terminal domain of NCOA4, thus preventing ferritinophagy (Tang et al., 2018). In addition, iron can promote the survival and growth of UPEC in host cells. (Table 1) Khasheii et al. (2016) demonstrated that free iron improves the growth rate of UPEC. Moreover, Bauckman et al. (2015) found that an increase in free iron caused by ferritinophagy also promotes the growth and survival of UPEC. UPEC may directly or indirectly drive ferritinophagy by depleting unstable iron pools and forcing UPEC to catabolize ferritin to provide iron for basic cellular functions. ATG16L1 protein is an autophagy-related protein. Studies have found that ATG16L1-deficient mice exhibit strong resistance to UPEC infection (Wang et al., 2012). When free iron is removed by an iron-chelating agent, the growth rate of UPEC is significantly inhibited (Brumbaugh et al., 2013). UPEC quiescent intracellular reservoirs in autophagosomes *in vivo* may lead to repeated infections as a result of UPEC following iron into autophagosomes, whereas UPEC is able to chelate iron through its siderophores.

**TABLE 1:** Treatment strategies of ferritinophagy-mediated ferroptosis in related diseases.

Disease		Key mechanism	Reference
Neurodegenerative diseases	Alzheimer's disease	Reduce free Fe^2+^ and lipid peroxides	Bellelli et al. (2016)
	Parkinson's disease	Reduce free Fe^2+^ and lipid peroxides	Jiang et al. (2017); Chen X et al. (2019)
	Huntington's disease	Inhibit Nrf2 degradation	McColgan and Tabrizi (2018)
Infectious disease	Urinary tract infections	Inhibit iron accumulation	Flores-Mireles et al. (2015); Khasheii et al. (2016)
Cardiocerebrovascular diseases	Dox-induced cardiac injury	Inhibit Nrf2 degradation Block lipid peroxidation	Conrad and Proneth (2019); Khamseekaew et al. (2017)
	Heart failure	Reduce Fe^2+^ and lipid peroxides	Li J et al. (2020); Ito et al. (2021)
	Atherosclerosis	Reduce Fe^2+^ and lipid peroxides	Zhu et al. (2018)
	Lung cancer	Reduce free Fe^2+^ and lipid peroxides Inactivation of GPX4	Zhang Y et al. (2021)
	Liver cancer	Activate Nrf2 pathway Increase free Fe^2+^ and lipid peroxides	Roh et al. (2017)
	Colorectal cancer	Suppress system Xc^−^ Increase free Fe^2+^ and lipid peroxides	Hasan et al. (2020)
Cancer	Glioblastoma	Block iron transport Increase free Fe^2+^ and lipid peroxides	Mancias et al. (2014): Zhang W et al. (2021)
	Breast cancer	Block iron transport; Increase free Fe^2+^ and lipid peroxides	Zhang et al. (2018); Zhu et al. (2017)
	Pancreas cancer	Suppress system Xc^−^ Block iron transport	Eling et al. (2015)
	Ovarian cancer	Inhibit system Xc^−^ activity	Rockfield et al. (2018)

Therefore, the regulation of ferritinophagy may have certain clinical value to reduce the persistence of UPEC and prevent the recurrence of UTI (Bauckman and Mysorekar, 2016). Interrupting ferritinophagy to control iron levels may provide a potentially therapeutic avenue to suppress UTI.

### Ferritinophagy and cardiocerebrovascular diseases

Both iron deficiency and iron overload are associated with cardiomyopathy an heart failure through complex mechanisms (Fang et al., 2020). Excessive iron accumulation in the heart leads to iron overload cardiomyopathy, the leading cause of death in patients with hemochromatosis (Sumneang et al., 2020). In a doxorubicin (Dox)-induced cardiomyopathy mouse model, RNA-seq analysis revealed differentially expressed genes, including HO-1 significantly upregulated (Conrad and Proneth, 2019). Iron overload was found in liver and kidney in HO-1 knockout mice, and the inhibition of HO-1 by zinc protoporphyrin IX reversed Dox-induced ferroptosis (Fang et al., 2019). Previous studies have reported that iron overload leads to cardiac mitochondrial dysfunction, which is primarily manifested by reduced mitochondrial respiration, increased mitochondrial ROS levels, and low mitochondrial swelling (Khamseekaew et al., 2017). Dox-induced cardiomyopathy mainly triggered a significant increase in the level of peroxidized phosphatidylethanolamine in mitochondria (Tadokoro et al., 2020). The above result further highlights the heart mitochondria in the role of lipid peroxide produced (Li et al., 2019; Jang et al., 2021). Studies have shown that lipopolysaccharide (LPS)-induced myocardial injury in a mouse sepsis model is closely associated with ferroptosis. Li N et al. (2020) found that after LPS treatment, the level of ferritinophagy was enhanced, and the mRNA expression and protein expression of sideroflexin 1 (SFXN1) were increased. A large amount of Fe^2+^ binds with SFXN1 and is transported to mitochondria, where a large amount of ROS is produced by the Fenton reaction and promotes the occurrence of ferroptosis. In in vitro experiments, LPS can increase the expression of NOCA4, promote ferritinophagy, and increase the release of free iron to cause lipid peroxidation, which may be one of the ferroptosis mechanisms that occurs in myocardial cells (Li J et al., 2020). The deletion of NCOA4 in mouse hearts improved cardiac function along with the attenuation of the upregulation of ferritinophagy-mediated ferritin degradation after stress overload (Li N et al., 2020; Ito et al., 2021). Ferrostatin-1 did not provide additional protection from pressure overload–induced cardiac remodeling in NCOA4^−/−^ mice, suggesting that iron-dependent cardiomyocyte death is downstream of NCOA4-mediated ferritinophagy. Toll-like receptor 4 and NADPH oxidase 4 knockdown can delay hyperactivated autophagy and ferroptosis (Chen X et al., 2019).

Abnormal lipid metabolism, oxidative stress, and inflammation are the main features of atherosclerosis (AS) (Zhu et al., 2018). The Nrf2-Keap1 pathway decreases ferroptosis associated with AS by maintaining cellular iron homeostasis, increasing the production of glutathione, GPX4, and NADPH. The overexpression of GPX4 in apolipoprotein E knockout (ApoE^−/−^) mice attenuates the upregulation of endothelial cell adhesion molecule and monocyte–endothelial cell adhesion, thereby inhibiting the development of AS (Guo et al., 2008). It has been reported that chronic iron overload could exacerbate the AS in ApoE^−/−^ mice by inducing oxidative stress and endothelial dysfunction (Habas and Shang, 2018; Marques et al., 2019). Zinc oxide nanoparticles (ZNOPs) exhibit various activities in biomedicine. They can be used as drug carriers but can be cytotoxic to vascular endothelial cells, thus aggravating vascular injury. Studies have shown that ZNOPs can lead to ferroptosis in vascular endothelial cells, whereas NCOA4 knockdown can reduce intracellular iron and lipid peroxidation. It was demonstrated that ZNOPs may lead to ferroptosis of endothelial cells through ferritinophagy (Qin et al., 2021). Iron overload induces endothelial dysfunction by enhancing oxidative and inflammatory responses of endothelial cells (Xu, 2019), revealing the potential relationship between ferritinophagy-induced ferroptosis and AS.

Ischemic stroke is a complex brain disease that is regulated by multiple pathways of cell death, including autophagy and ferroptosis. However, the potential association between autophagy and ferroptosis during ischemic stroke has not been determined. Since the destruction of iron homeostasis and autophagy defects coexist in ischemic stroke and autophagy promotes the clearance of toxic proteins to maintain cell homeostasis, we postulate that abnormal autophagy is likely to mediate the degradation of ferritin, thus promoting iron overload and ferroptosis during ischemic stroke (Liu et al., 2020). NCOA4 may play a role in FTH1 conversion in the brain (Santana-Codina et al., 2019). High serum ferritin content is also associated with poor prognosis during ischemic stroke (García-Yébenes et al., 2012), suggesting that ferritin plays an important role in mediating iron storage and release. TfR1 is a major receptor involved in iron transport to the brain, which plays an important role in maintaining iron homeostasis and regulating iron atonia. Thus, the inhibition of ferroptosis by inhibiting free Fe^2+^, and reducing ROS accumulation and lipid peroxidation may provide a new approach to the treatment of ischemic stroke.

### Ferritinophagy and cancer

Normal iron metabolism is a marker of abnormal expression in many cancers. Intracellular ferritin imbalance is closely associated with the development of cancer, and there is increasing evidence that ferritinophagy and ferroptosis play an important role in tumor therapy (Zhang et al., 2018, Zhu et al., 2017). NCOA4 may be relevant to carcinogenesis, and the intersection of ferritinophagy and ferroptosis pathways may represent a therapeutic approach (Shaw et al., 2001). A positive correlation between NCOA4 mRNA and NCOA4α protein levels and transformation has been reported in ovarian carcinoma. The expression and function of NCOA4 isoforms have been reported in transformed endometriotic and malignant ovarian cancer cells (Rockfield et al., 2018). The overexpression of several oncogenes (MYC, H-Ras, and p53 inactivation) in normal endometriotic cells to induce transformation resulted in an upregulation of NCOA4α and NCOA4β expression. NCOA4α knockdown in transformed cells decreased survival, whereas NCOA4β overexpression decreased colony formation. In contrast, in studies of prostate cancer, NCOA4α acts as a tumor suppressor, whereas NCOA4β expression correlated with proliferation and invasion. The prostate cancer cell proliferation and invasion was stimulated by the androgen receptor coactivator, androgen receptor-associated protein 70 (Peng et al., 2008). NCOA4-mediated ferritinophagy promotes ferroptosis induced by erastin, but not by RSL3 in HeLa cells. Ferritinophagy is required for the induction of ferroptosis by the bromodomain protein, bromodomain-containing protein 4 inhibitor (+)-JQ1, in cancer cells. Low expression of the ferritinophagy-related NCOA4 gene was related to unfavorable outcomes and defective immune cell infiltration in clear cell renal carcinoma. Lipid peroxidation–mediated ferroptosis has recently attracted widespread attention as a potential therapeutic strategy for cancer. Zeta1 (COPZ1), the complex subunit of the coat protein, is a putative therapeutic target for glioblastoma, which can significantly affect iron metabolism and tumor prognosis. The COPZ1-NCOA4-FTH1 axis may represent a new target for the treatment of glioblastoma (Zhang W et al., 2021). Pancreatic cancer cell lines have increased NCOA4 levels and a corresponding high flux through the ferritinophagy pathway. Quantitative proteomics identifies NCOA4 as the cargo receptor mediating ferritinophagy (Mancias et al., 2014). Direct evidence for the role of NCOA4 in modulating ferroptotic cell death in pancreatic cancer cells was provided by Yang *et al.* who showed that artesunate-mediated ferroptotic cell death is attenuated by NCOA4 depletion (Eling et al., 2015).

System Xc^−^ is the main active site for erastin, which was first screened for its ability to kill tumor cells with almost no toxicity against homologous normal cells with Ras mutations (Wang et al., 2020). SLC7A11 is overexpressed in various malignant tumors and is closely related to the growth, prognosis, metastasis, and treatment of malignant tumors including breast, ovarian, liver, and lung cancers. RNA-binding protein (RBP) plays a role in the regulation of gene expression. Yang *et al.* found that RBP, RBMS1, affects ferroptosis in lung cancer by regulating the translation of SLC7A11. NTP can lead to the downregulation of RBMS1 in radiotherapy-resistant cells, thus reducing the expression of SLC7A11 and promoting the ferroptosis. This renders radiotherapy-resistant lung cancer cells sensitive to radiotherapy and provides a new treatment for lung cancer patients (Zhang Y et al., 2021). Xue *et al.* demonstrated an important role of micronutrient iron in colorectal cancer growth. Conversely, ferritinophagy is not required for colon cancer cell growth. Compared with other tissues, the gut is a major tissue that is essential for normal dietary iron absorption. Therefore, it contains several mechanisms that regulate apical iron transport and basolateral outflow. Hasan et al. (2020) demonstrated a marginal role for ferritinophagy in growth under normal and cytotoxic conditions in colon cancer cells, as well as a possible compensatory mechanism in response to ferroptosis. This study demonstrated that ferritinophagy is not required for the basal growth of colorectal cancer (CRC)–derived cells. The main reason is that an increased ability of colon cancer cells to uptake iron from the extracellular environment may compensate for the loss of NCOA4 function through the exclusive use of extracellular iron for survival. In addition, an increase in Ftn protein expression may also serve as a protective mechanism by sequestering the iron needed for cell death and ferroptosis into Ftn. CRC pathogenesis is dependent upon iron. TfR1 expression has also been found to be high in CRC tissues and CRC-derived cell lines. Its downregulation promotes cancer progression, which underscores the role of transferrin-bound iron uptake in tumor growth (Cui et al., 2019).

Intracellular ROS imbalance–mediated oxidative stress defense is also closely related to drug resistance in tumors. Currently, acquired resistance to chemotherapy is a major cause of tumor progression. Iron overload in tumor cells catalyzes the production of ROS and meets the needs of cell proliferation to a certain extent; however, when tumor cells are exposed to chemotherapeutic drugs, ROS production is induced (Galadari et al., 2017). The sensitivity of K562 leukemia cells to doxorubicin may be increased by treating it with the iron-chelating agent, deferoxamine (DFO), to inhibit iron overload. DFO treatment of cervical cancer cells enhances their sensitivity to oxaliplatin, providing direct evidence that iron overload is involved in chemotherapeutic drug resistance (Chen et al., 2016). DFO treatment of different types of ovarian cancer cells significantly increases intracellular iron ions and ROS (Watson, 2013). Recent studies showed that the nuclear transcription factor, Nrf2, is closely associated with the regulation of ferroptosis and the inhibition of the Nrf2-Keap1 signaling pathway activation promotes ferroptosis and reverses drug resistance in cisplatin-resistant head and neck tumors (Roh et al., 2017). Sorafenib, a targeted therapy for liver cancer, is a strong inducer of ferroptosis. The activation of the Nrf2 pathway in liver cancer cells can upregulate the expression of metallothionein-1G (MT-1G) and promotes sorafenib drug resistance by inhibiting ferroptosis (Roh et al., 2017). Therefore, triggering ferroptosis in cancer with high autophagy levels may reveal a potential vulnerability. One possible therapeutic strategy is to increase iron flux through the ferritinophagy pathway, leading to increased labile iron and ROS and sensitizing cancer cells to ferroptosis-inducing agents. Thus, inducing autophagy-dependent ferroptosis may represent a new antitumor strategy.

## Conclusion and perspective

Ferritinophagy is an important mechanism that regulates iron levels *in vivo*. It has been demonstrated that ferritinophagy is closely related to nervous system diseases, tumors, and infectious diseases. Although the molecular mechanism of ferritinophagy remains undefined, the inhibition of ferritinophagy can prevent and reverse various diseases. Further research on the mechanism of ferritinophagy is expected to provide a theoretical basis for the treatment of related diseases in the future.

## References

[B1] Bai Y. Meng L. Han L. Jia Y. Zhao Y. Gao H. (2019). Lipid storage and lipophagy regulates ferroptosis. Biochem. Biophys. Res. Commun. 508, 997–1003. 10.1016/j.bbrc.2018.12.039 PubMed Abstract | 10.1016/j.bbrc.2018.12.039 | Google Scholar 30545638

[B2] Baksi S. Singh N. (2017). α-Synuclein impairs ferritinophagy in the retinal pigment epithelium: Implications for retinal iron dyshomeostasis in Parkinson's disease. Sci. Rep. 7, 12843. 10.1038/s41598-017-12862-x PubMed Abstract | 10.1038/s41598-017-12862-x | Google Scholar 28993630 PMC5634503

[B3] Bauckman K. A. Mysorekar I. U. (2016). Ferritinophagy drives uropathogenic *Escherichia coli* persistence in bladder epithelial cells. Autophagy 12, 850–863. 10.1080/15548627.2016.1160176 PubMed Abstract | 10.1080/15548627.2016.1160176 | Google Scholar 27002654 PMC4854542

[B4] Bauckman K. A. Owusu-Boaitey N. Mysorekar I. U. (2015). Selective autophagy: xenophagy. Methods 75, 120–127. 10.1016/j.ymeth.2014.12.005 PubMed Abstract | 10.1016/j.ymeth.2014.12.005 | Google Scholar 25497060 PMC4355331

[B5] Bayeva M. Chang H. C. Wu R. Ardehali H. (2013). When less is more: novel mechanisms of iron conservation. Trends Endocrinol. Metab. 24, 569–577. 10.1016/j.tem.2013.07.003 PubMed Abstract | 10.1016/j.tem.2013.07.003 | Google Scholar 23948590 PMC4720524

[B6] Bellelli R. Federico G. Matte A. Colecchia D. Iolascon A. Chiariello M. (2016). NCOA4 deficiency impairs systemic iron homeostasis. Cell Rep. 14, 411–421. 10.1016/j.celrep.2015.12.065 PubMed Abstract | 10.1016/j.celrep.2015.12.065 | Google Scholar 26776506

[B7] Biasiotto G. Di Lorenzo D. Archetti S. Zanella I. (2016). Iron and neurodegeneration: Is ferritinophagy the link? Mol. Neurobiol. 53, 5542–5574. 10.1007/s12035-015-9473-y PubMed Abstract | 10.1007/s12035-015-9473-y | Google Scholar 26468157

[B8] Brumbaugh A. R. Smith S. N. Mobley H. L. (2013). Immunization with the yersiniabactin receptor, FyuA, protects against pyelonephritis in a murine model of urinary tract infection. Infect. Immun. 81, 3309–3316. 10.1128/iai.00470-13 PubMed Abstract | 10.1128/iai.00470-13 | Google Scholar 23798537 PMC3754202

[B9] Bulvik B. E. Berenshtein E. Meyron-Holtz E. G. Konijn A. M. Chevion M. (2012). Cardiac protection by preconditioning is generated via an iron-signal created by proteasomal degradation of iron proteins. PLoS One 7, e48947. 10.1371/journal.pone.0048947 PubMed Abstract | 10.1371/journal.pone.0048947 | Google Scholar 23155431 PMC3498359

[B10] Campanella A. Rovelli E. Santambrogio P. Cozzi A. Taroni F. Levi S. (2009). Mitochondrial ferritin limits oxidative damage regulating mitochondrial iron availability: hypothesis for a protective role in friedreich ataxia. Hum. Mol. Genet. 18, 1–11. 10.1093/hmg/ddn308 PubMed Abstract | 10.1093/hmg/ddn308 | Google Scholar 18815198 PMC3298861

[B11] Chen S. J. Kuo C. C. Pan H. Y. Tsou T. C. Yeh S. C. Chang J. Y. (2016). Desferal regulates hCtr1 and transferrin receptor expression through Sp1 and exhibits synergistic cytotoxicity with platinum drugs in oxaliplatin-resistant human cervical cancer cells *in vitro* and *in vivo* . Oncotarget 7, 49310–49321. 10.18632/oncotarget.10336 PubMed Abstract | 10.18632/oncotarget.10336 | Google Scholar 27384479 PMC5226510

[B12] Chen X. Yu C. Kang R. Tang D. (2020). Iron metabolism in ferroptosis. Front. Cell Dev. Biol. 8, 590226. 10.3389/fcell.2020.590226 PubMed Abstract | 10.3389/fcell.2020.590226 | Google Scholar 33117818 PMC7575751

[B13] Chen L. L. Huang Y. J. Cui J. T. Song N. Xie J. (2019). Iron dysregulation in Parkinson's disease: Focused on the autophagy-lysosome pathway. ACS Chem. Neurosci. 10, 863–871. 10.1021/acschemneuro.8b00390 PubMed Abstract | 10.1021/acschemneuro.8b00390 | Google Scholar 30590010

[B14] Chen X. Xu S. Zhao C. Liu B. (2019). Role of TLR4/NADPH oxidase 4 pathway in promoting cell death through autophagy and ferroptosis during heart failure. Biochem. Biophys. Res. Commun. 516, 37–43. 10.1016/j.bbrc.2019.06.015 PubMed Abstract | 10.1016/j.bbrc.2019.06.015 | Google Scholar 31196626

[B15] Chiang S. K. Chen S. E. Chang L. C. (2018). A dual role of heme oxygenase-1 in cancer cells. Int. J. Mol. Sci. 20, E39. 10.3390/ijms20010039 PubMed Abstract | 10.3390/ijms20010039 | Google Scholar 30583467 PMC6337503

[B16] Choi Y. K. Kim Y. M. (2022). Beneficial and detrimental roles of heme oxygenase-1 in the neurovascular system. Int. J. Mol. Sci. 23, 7041. 10.3390/ijms23137041 PubMed Abstract | 10.3390/ijms23137041 | Google Scholar 35806040 PMC9266949

[B17] Conrad M. Proneth B. (2019). Broken hearts: Iron overload, ferroptosis and cardiomyopathy. Cell Res. 29, 263–264. 10.1038/s41422-019-0150-y PubMed Abstract | 10.1038/s41422-019-0150-y | Google Scholar 30809018 PMC6461867

[B18] Cui C. Cheng X. Yan L. Ding H. Guan X. Zhang W. (2019). Downregulation of TfR1 promotes progression of colorectal cancer via the JAK/STAT pathway. Cancer Manag. Res. 11, 6323–6341. 10.2147/cmar.S198911 PubMed Abstract | 10.2147/cmar.S198911 | Google Scholar 31372038 PMC6628123

[B19] Doll S. Conrad M. (2017). Iron and ferroptosis: A still ill-defined liaison. IUBMB Life 69, 423–434. 10.1002/iub.1616 PubMed Abstract | 10.1002/iub.1616 | Google Scholar 28276141

[B20] Dong H. Qiang Z. Chai D. Peng J. Xia Y. Hu R. (2020). Nrf2 inhibits ferroptosis and protects against acute lung injury due to intestinal ischemia reperfusion via regulating SLC7A11 and HO-1. Aging (Albany NY) 12, 12943–12959. 10.18632/aging.103378 PubMed Abstract | 10.18632/aging.103378 | Google Scholar 32601262 PMC7377827

[B21] Drummond G. S. Baum J. Greenberg M. Lewis D. Abraham N. G. (2019). HO-1 overexpression and underexpression: Clinical implications. Arch. Biochem. Biophys. 673, 108073. 10.1016/j.abb.2019.108073 PubMed Abstract | 10.1016/j.abb.2019.108073 | Google Scholar 31425676 PMC6748652

[B22] Eling N. Reuter L. Hazin J. Hamacher-Brady A. Brady N. R. (2015). Identification of artesunate as a specific activator of ferroptosis in pancreatic cancer cells. Oncoscience 2, 517–532. 10.18632/oncoscience.160 PubMed Abstract | 10.18632/oncoscience.160 | Google Scholar 26097885 PMC4468338

[B23] Facchinetti M. M. (2020). Heme-Oxygenase-1. Antioxid. Redox Signal. 32, 1239–1242. 10.1089/ars.2020.8065 PubMed Abstract | 10.1089/ars.2020.8065 | Google Scholar 32148070

[B24] Fang X. Wang H. Han D. Xie E. Yang X. Wei J. (2019). Ferroptosis as a target for protection against cardiomyopathy. Proc. Natl. Acad. Sci. U. S. A. 116, 2672–2680. 10.1073/pnas.1821022116 PubMed Abstract | 10.1073/pnas.1821022116 | Google Scholar 30692261 PMC6377499

[B25] Fang X. Cai Z. Wang H. Han D. Cheng Q. Zhang P. (2020). Loss of cardiac ferritin H facilitates cardiomyopathy via slc7a11-mediated ferroptosis. Circ. Res. 127, 486–501. 10.1161/circresaha.120.316509 PubMed Abstract | 10.1161/circresaha.120.316509 | Google Scholar 32349646

[B26] Fang X. Ardehali H. Min J. Wang F. (2022). The molecular and metabolic landscape of iron and ferroptosis in cardiovascular disease. Nat. Rev. Cardiol. 1–17. 10.1038/s41569-022-00735-4 10.1038/s41569-022-00735-4 | Google Scholar PMC9252571 35788564

[B27] Febbraro F. Giorgi M. Caldarola S. Loreni F. Romero-Ramos M. (2012). α-Synuclein expression is modulated at the translational level by iron. Neuroreport 23, 576–580. 10.1097/WNR.0b013e328354a1f0 PubMed Abstract | 10.1097/WNR.0b013e328354a1f0 | Google Scholar 22581044

[B28] Feng H. Schorpp K. Jin J. Yozwiak C. E. Hoffstrom B. G. Decker A. M. (2020). Transferrin receptor is a specific ferroptosis marker. Cell Rep. 30, 3411–3423. 10.1016/j.celrep.2020.02.049 PubMed Abstract | 10.1016/j.celrep.2020.02.049 | Google Scholar 32160546 PMC7172030

[B29] Feng J. Li C. Xu R. Li Y. Hou Q. Feng R. (2020). DpdtC-induced EMT inhibition in MGC-803 cells was partly through ferritinophagy-mediated ROS/p53 pathway. Oxid. Med. Cell. Longev. 2020, 9762390. 10.1155/2020/9762390 PubMed Abstract | 10.1155/2020/9762390 | Google Scholar 32256964 PMC7091554

[B30] Flores-Mireles A. L. Walker J. N. Caparon M. Hultgren S. J. (2015). Urinary tract infections: epidemiology, mechanisms of infection and treatment options. Nat. Rev. Microbiol. 13, 269–284. 10.1038/nrmicro3432 PubMed Abstract | 10.1038/nrmicro3432 | Google Scholar 25853778 PMC4457377

[B31] Fuhrmann D. C. Mondorf A. Beifuß J. Jung M. Brüne B. (2020). Hypoxia inhibits ferritinophagy, increases mitochondrial ferritin, and protects from ferroptosis. Redox Biol. 36, 101670. 10.1016/j.redox.2020.101670 PubMed Abstract | 10.1016/j.redox.2020.101670 | Google Scholar 32810738 PMC7452134

[B32] Galadari S. Rahman A. Pallichankandy S. Thayyullathil F. (2017). Reactive oxygen species and cancer paradox: To promote or to suppress? Free Radic. Biol. Med. 104, 144–164. 10.1016/j.freeradbiomed.2017.01.004 PubMed Abstract | 10.1016/j.freeradbiomed.2017.01.004 | Google Scholar 28088622

[B33] Gammella E. Recalcati S. Cairo G. (2016). Dual role of ROS as signal and stress agents: Iron tips the balance in favor of toxic effects. Oxid. Med. Cell. Longev. 2016, 8629024. 10.1155/2016/8629024 PubMed Abstract | 10.1155/2016/8629024 | Google Scholar 27006749 PMC4783558

[B34] Gao M. Monian P. Pan Q. Zhang W. Xiang J. Jiang X. (2016). Ferroptosis is an autophagic cell death process. Cell Res. 26, 1021–1032. 10.1038/cr.2016.95 PubMed Abstract | 10.1038/cr.2016.95 | Google Scholar 27514700 PMC5034113

[B35] García-Yébenes I. Sobrado M. Moraga A. Zarruk J. G. Romera V. G. Pradillo J. M. (2012). Iron overload, measured as serum ferritin, increases brain damage induced by focal ischemia and early reperfusion. Neurochem. Int. 61, 1364–1369. 10.1016/j.neuint.2012.09.014 PubMed Abstract | 10.1016/j.neuint.2012.09.014 | Google Scholar 23036361

[B36] Griffiths R. E. Kupzig S. Cogan N. Mankelow T. J. Betin V. M. Trakarnsanga K. (2012). The ins and outs of human reticulocyte maturation: autophagy and the endosome/exosome pathway. Autophagy 8, 1150–1151. 10.4161/auto.20648 PubMed Abstract | 10.4161/auto.20648 | Google Scholar 22659916 PMC3429555

[B37] Guo Z. Ran Q. Roberts L. J. 2nd Zhou L. Richardson A. Sharan C. (2008). Suppression of atherogenesis by overexpression of glutathione peroxidase-4 in apolipoprotein E-deficient mice. Free Radic. Biol. Med. 44, 343–352. 10.1016/j.freeradbiomed.2007.09.009 PubMed Abstract | 10.1016/j.freeradbiomed.2007.09.009 | Google Scholar 18215741 PMC2245803

[B38] Habas K. Shang L. (2018). Alterations in intercellular adhesion molecule 1 (ICAM-1) and vascular cell adhesion molecule 1 (VCAM-1) in human endothelial cells. Tissue Cell 54, 139–143. 10.1016/j.tice.2018.09.002 PubMed Abstract | 10.1016/j.tice.2018.09.002 | Google Scholar 30309503

[B39] Hara Y. Yanatori I. Tanaka A. Kishi F. Lemasters J. J. Nishina S. (2020). Iron loss triggers mitophagy through induction of mitochondrial ferritin. EMBO Rep. 21, e50202. 10.15252/embr.202050202 PubMed Abstract | 10.15252/embr.202050202 | Google Scholar 32975364 PMC7645172

[B40] Hasan M. Reddy S. M. Das N. K. (2020). Ferritinophagy is not required for colon cancer cell growth. Cell Biol. Int. 44, 2307–2314. 10.1002/cbin.11439 PubMed Abstract | 10.1002/cbin.11439 | Google Scholar 32767706

[B41] Hou W. Xie Y. Song X. Sun X. Lotze M. T. Zeh H. J. (2016). Autophagy promotes ferroptosis by degradation of ferritin. Autophagy 12, 1425–1428. 10.1080/15548627.2016.1187366 PubMed Abstract | 10.1080/15548627.2016.1187366 | Google Scholar 27245739 PMC4968231

[B42] Huang Y. Yang Y. Xu Y. Ma Q. Guo F. Zhao Y. (2021). Nrf2/HO-1 Axis regulates the angiogenesis of gastric cancer via targeting VEGF. Cancer Manag. Res. 13, 3155–3169. 10.2147/cmar.S292461 PubMed Abstract | 10.2147/cmar.S292461 | Google Scholar 33889021 PMC8055645

[B43] Ito J. Omiya S. Rusu M. C. Ueda H. Murakawa T. Tanada Y. (2021). Iron derived from autophagy-mediated ferritin degradation induces cardiomyocyte death and heart failure in mice. Elife 10, e62174. 10.7554/eLife.62174 PubMed Abstract | 10.7554/eLife.62174 | Google Scholar 33526170 PMC7853718

[B44] Jang S. Chapa-Dubocq X. R. Tyurina Y. Y. St Croix C. M. Kapralov A. A. Tyurin V. A. (2021). Elucidating the contribution of mitochondrial glutathione to ferroptosis in cardiomyocytes. Redox Biol. 45, 102021. 10.1016/j.redox.2021.102021 PubMed Abstract | 10.1016/j.redox.2021.102021 | Google Scholar 34102574 PMC8187237

[B45] Jiang H. Wang J. Rogers J. Xie J. (2017). Brain iron metabolism dysfunction in Parkinson's disease. Mol. Neurobiol. 54, 3078–3101. 10.1007/s12035-016-9879-1 PubMed Abstract | 10.1007/s12035-016-9879-1 | Google Scholar 27039308

[B46] Jiang X. Stockwell B. R. Conrad M. (2021). Ferroptosis: mechanisms, biology and role in disease. Nat. Rev. Mol. Cell Biol. 22, 266–282. 10.1038/s41580-020-00324-8 PubMed Abstract | 10.1038/s41580-020-00324-8 | Google Scholar 33495651 PMC8142022

[B47] Kakhlon O. Cabantchik Z. I. (2002). The labile iron pool: characterization, measurement, and participation in cellular processes(1). Free Radic. Biol. Med. 33, 1037–1046. 10.1016/s0891-5849(02)01006-7 PubMed Abstract | 10.1016/s0891-5849(02)01006-7 | Google Scholar 12374615

[B48] Khamseekaew J. Kumfu S. Wongjaikam S. Kerdphoo S. Jaiwongkam T. Srichairatanakool S. (2017). Effects of iron overload, an iron chelator and a T-Type calcium channel blocker on cardiac mitochondrial biogenesis and mitochondrial dynamics in thalassemic mice. Eur. J. Pharmacol. 799, 118–127. 10.1016/j.ejphar.2017.02.015 PubMed Abstract | 10.1016/j.ejphar.2017.02.015 | Google Scholar 28192097

[B49] Khasheii B. Anvari S. Jamalli A. (2016). Frequency evaluation of genes encoding siderophores and the effects of different concentrations of Fe ions on growth rate of uropathogenic *Escherichia coli* . Iran. J. Microbiol. 8, 359–365. PubMed Abstract | Google Scholar 28491245 PMC5420389

[B50] Lawson D. M. Artymiuk P. J. Yewdall S. J. Smith J. M. Livingstone J. C. Treffry A. (1991). Solving the structure of human H ferritin by genetically engineering intermolecular crystal contacts. Nature 349, 541–544. 10.1038/349541a0 PubMed Abstract | 10.1038/349541a0 | Google Scholar 1992356

[B51] Li W. Feng G. Gauthier J. M. Lokshina I. Higashikubo R. Evans S. (2019). Ferroptotic cell death and TLR4/Trif signaling initiate neutrophil recruitment after heart transplantation. J. Clin. Invest. 129, 2293–2304. 10.1172/jci126428 PubMed Abstract | 10.1172/jci126428 | Google Scholar 30830879 PMC6546457

[B52] Li H. B. Zhang X. Z. Sun Y. Zhou Q. Song J. N. Hu Z. F. (2020). HO-1/PINK1 regulated mitochondrial fusion/fission to inhibit pyroptosis and attenuate septic acute kidney injury. Biomed. Res. Int. 2020, 2148706. 10.1155/2020/2148706 PubMed Abstract | 10.1155/2020/2148706 | Google Scholar 33145342 PMC7599399

[B53] Li J. Cao F. Yin H. L. Huang Z. J. Lin Z. T. Mao N. (2020). Ferroptosis: past, present and future. Cell Death Dis. 11, 88. 10.1038/s41419-020-2298-2 PubMed Abstract | 10.1038/s41419-020-2298-2 | Google Scholar 32015325 PMC6997353

[B54] Li N. Wang W. Zhou H. Wu Q. Duan M. Liu C. (2020). Ferritinophagy-mediated ferroptosis is involved in sepsis-induced cardiac injury. Free Radic. Biol. Med. 160, 303–318. 10.1016/j.freeradbiomed.2020.08.009 PubMed Abstract | 10.1016/j.freeradbiomed.2020.08.009 | Google Scholar 32846217

[B55] Liu J. Guo Z. N. Yan X. L. Huang S. Ren J. X. Luo Y. (2020). Crosstalk between autophagy and ferroptosis and its putative role in ischemic stroke. Front. Cell. Neurosci. 14, 577403. 10.3389/fncel.2020.577403 PubMed Abstract | 10.3389/fncel.2020.577403 | Google Scholar 33132849 PMC7566169

[B56] Liu X. J. Lv Y. F. Cui W. Z. Li Y. Liu Y. Xue Y. T. (2021). Icariin inhibits hypoxia/reoxygenation-induced ferroptosis of cardiomyocytes via regulation of the Nrf2/HO-1 signaling pathway. FEBS Open Bio 11, 2966–2976. 10.1002/2211-5463.13276 PubMed Abstract | 10.1002/2211-5463.13276 | Google Scholar PMC8564343 34407320

[B57] Ma H. Wang X. Zhang W. Li H. Zhao W. Sun J. (2020). Melatonin suppresses ferroptosis induced by high glucose via activation of the Nrf2/HO-1 signaling pathway in type 2 diabetic osteoporosis. Oxid. Med. Cell. Longev. 2020, 9067610. 10.1155/2020/9067610 PubMed Abstract | 10.1155/2020/9067610 | Google Scholar 33343809 PMC7732386

[B58] Ma L. L. Sun L. Wang Y. X. Sun B. H. Li Y. F. Jin Y. L. (2022). Association between HO-1 gene promoter polymorphisms and diseases (Review). Mol. Med. Rep. 25, 29. 10.3892/mmr.2021.12545 PubMed Abstract | 10.3892/mmr.2021.12545 | Google Scholar 34841438 PMC8669660

[B59] Mancias J. D. Wang X. Gygi S. P. Harper J. W. Kimmelman A. C. (2014). Quantitative proteomics identifies NCOA4 as the cargo receptor mediating ferritinophagy. Nature 509, 105–109. 10.1038/nature13148 PubMed Abstract | 10.1038/nature13148 | Google Scholar 24695223 PMC4180099

[B60] Mancias J. D. Pontano Vaites L. Nissim S. Biancur D. E. Kim A. J. Wang X. (2015). Ferritinophagy via NCOA4 is required for erythropoiesis and is regulated by iron dependent HERC2-mediated proteolysis. Elife 4. 10.7554/eLife.10308 PubMed Abstract | 10.7554/eLife.10308 | Google Scholar PMC4592949 26436293

[B61] Marques V. B. Leal M. A. S. Mageski J. G. A. Fidelis H. G. Nogueira B. V. Vasquez E. C. (2019). Chronic iron overload intensifies atherosclerosis in apolipoprotein E deficient mice: Role of oxidative stress and endothelial dysfunction. Life Sci. 233, 116702. 10.1016/j.lfs.2019.116702 PubMed Abstract | 10.1016/j.lfs.2019.116702 | Google Scholar 31356905

[B62] McColgan P. Tabrizi S. J. (2018). Huntington's disease: a clinical review. Eur. J. Neurol. 25, 24–34. 10.1111/ene.13413 PubMed Abstract | 10.1111/ene.13413 | Google Scholar 28817209

[B63] Mena N. P. Urrutia P. J. Lourido F. Carrasco C. M. Núñez M. T. (2015). Mitochondrial iron homeostasis and its dysfunctions in neurodegenerative disorders. Mitochondrion 21, 92–105. 10.1016/j.mito.2015.02.001 PubMed Abstract | 10.1016/j.mito.2015.02.001 | Google Scholar 25667951

[B64] Moroishi T. Yamauchi T. Nishiyama M. Nakayama K. I. (2014). HERC2 targets the iron regulator FBXL5 for degradation and modulates iron metabolism. J. Biol. Chem. 289, 16430–16441. 10.1074/jbc.M113.541490 PubMed Abstract | 10.1074/jbc.M113.541490 | Google Scholar 24778179 PMC4047410

[B65] Peng Y. Li C. X. Chen F. Wang Z. Ligr M. Melamed J. (2008). Stimulation of prostate cancer cellular proliferation and invasion by the androgen receptor co-activator ARA70. Am. J. Pathol. 172, 225–235. 10.2353/ajpath.2008.070065 PubMed Abstract | 10.2353/ajpath.2008.070065 | Google Scholar 18156210 PMC2189610

[B66] Qin X. Zhang J. Wang B. Xu G. Yang X. Zou Z. (2021). Ferritinophagy is involved in the zinc oxide nanoparticles-induced ferroptosis of vascular endothelial cells. Autophagy 17, 4266–4285. 10.1080/15548627.2021.1911016 PubMed Abstract | 10.1080/15548627.2021.1911016 | Google Scholar 33843441 PMC8726675

[B67] Rockfield S. Flores I. Nanjundan M. (2018). Expression and function of nuclear receptor coactivator 4 isoforms in transformed endometriotic and malignant ovarian cells. Oncotarget 9, 5344–5367. 10.18632/oncotarget.23747 PubMed Abstract | 10.18632/oncotarget.23747 | Google Scholar 29435183 PMC5797054

[B68] Roh J. L. Kim E. H. Jang H. Shin D. (2017). Nrf2 inhibition reverses the resistance of cisplatin-resistant head and neck cancer cells to artesunate-induced ferroptosis. Redox Biol. 11, 254–262. 10.1016/j.redox.2016.12.010 PubMed Abstract | 10.1016/j.redox.2016.12.010 | Google Scholar 28012440 PMC5198738

[B69] Rouault T. A. (2006). The role of iron regulatory proteins in mammalian iron homeostasis and disease. Nat. Chem. Biol. 2, 406–414. 10.1038/nchembio807 PubMed Abstract | 10.1038/nchembio807 | Google Scholar 16850017

[B70] Santana-Codina N. Gableske S. Quiles del Rey M. Małachowska B. Jedrychowski M. P. Biancur D. E. (2019). NCOA4 maintains murine erythropoiesis via cell autonomous and non-autonomous mechanisms. Haematologica 104, 1342–1354. 10.3324/haematol.2018.204123 PubMed Abstract | 10.3324/haematol.2018.204123 | Google Scholar 30630985 PMC6601094

[B71] Shaw P. A. Rittenberg P. V. Brown T. J. (2001). Activation of androgen receptor-associated protein 70 (ARA70) mRNA expression in ovarian cancer. Gynecol. Oncol. 80, 132–138. 10.1006/gyno.2000.6068 PubMed Abstract | 10.1006/gyno.2000.6068 | Google Scholar 11161850

[B72] Song N. Xie J. (2018). Iron, dopamine, and α-synuclein interactions in at-risk dopaminergic neurons in Parkinson's disease. Neurosci. Bull. 34, 382–384. 10.1007/s12264-018-0209-7 PubMed Abstract | 10.1007/s12264-018-0209-7 | Google Scholar 29380248 PMC5856725

[B73] Song Y. Wang B. Zhu X. Hu J. Sun J. Xuan J. (2021). Human umbilical cord blood-derived MSCs exosome attenuate myocardial injury by inhibiting ferroptosis in acute myocardial infarction mice. Cell Biol. Toxicol. 37, 51–64. 10.1007/s10565-020-09530-8 PubMed Abstract | 10.1007/s10565-020-09530-8 | Google Scholar 32535745

[B74] Stockwell B. R. Friedmann Angeli J. P. Bayir H. Bush A. I. Conrad M. Dixon S. J. (2017). Ferroptosis: A regulated cell death nexus linking metabolism, redox biology, and disease. Cell 171, 273–285. 10.1016/j.cell.2017.09.021 PubMed Abstract | 10.1016/j.cell.2017.09.021 | Google Scholar 28985560 PMC5685180

[B75] Sumneang N. Siri-Angkul N. Kumfu S. Chattipakorn S. C. Chattipakorn N. (2020). The effects of iron overload on mitochondrial function, mitochondrial dynamics, and ferroptosis in cardiomyocytes. Arch. Biochem. Biophys. 680, 108241. 10.1016/j.abb.2019.108241 PubMed Abstract | 10.1016/j.abb.2019.108241 | Google Scholar 31891670

[B76] Tadokoro T. Ikeda M. Ide T. Deguchi H. Ikeda S. Okabe K. (2020). Mitochondria-dependent ferroptosis plays a pivotal role in doxorubicin cardiotoxicity. JCI Insight 5, 132747. 10.1172/jci.insight.132747 PubMed Abstract | 10.1172/jci.insight.132747 | Google Scholar 32376803 PMC7253028

[B77] Tang M. Chen Z. Wu D. Chen L. (2018). Ferritinophagy/ferroptosis: Iron-related newcomers in human diseases. J. Cell. Physiol. 233, 9179–9190. 10.1002/jcp.26954 PubMed Abstract | 10.1002/jcp.26954 | Google Scholar 30076709

[B78] Tian Y. Lu J. Hao X. Li H. Zhang G. Liu X. (2020). FTH1 inhibits ferroptosis through ferritinophagy in the 6-OHDA model of Parkinson's disease. Neurotherapeutics 17, 1796–1812. 10.1007/s13311-020-00929-z PubMed Abstract | 10.1007/s13311-020-00929-z | Google Scholar 32959272 PMC7851296

[B79] Ursini F. Maiorino M. (2020). Lipid peroxidation and ferroptosis: The role of GSH and GPx4. Free Radic. Biol. Med. 152, 175–185. 10.1016/j.freeradbiomed.2020.02.027 PubMed Abstract | 10.1016/j.freeradbiomed.2020.02.027 | Google Scholar 32165281

[B80] Van der Riet F. D. Sayed A. R. Barnard B. J. van Tonder E. M. Crouse W. J. (1985). Arthropod-borne virus zoonosis surveillance in the cape province: 1. Prospective serological investigations for virus activity in the beaufort west and middelburg districts during 1981. J. S. Afr. Vet. Assoc. 56, 25–29. PubMed Abstract | Google Scholar 2987499

[B81] Wang C. Symington J. W. Mysorekar I. U. (2012). ATG16L1 and pathogenesis of urinary tract infections. Autophagy 8, 1693–1694. 10.4161/auto.21600 PubMed Abstract | 10.4161/auto.21600 | Google Scholar 22874553 PMC3494604

[B82] Wang Y. Q. Chang S. Y. Wu Q. Gou Y. J. Jia L. Cui Y. M. (2016). The protective role of mitochondrial ferritin on erastin-induced ferroptosis. Front. Aging Neurosci. 8, 308. 10.3389/fnagi.2016.00308 PubMed Abstract | 10.3389/fnagi.2016.00308 | Google Scholar 28066232 PMC5167726

[B83] Wang Y. Chen Q. Shi C. Jiao F. Gong Z. (2019). Mechanism of glycyrrhizin on ferroptosis during acute liver failure by inhibiting oxidative stress. Mol. Med. Rep. 20, 4081–4090. 10.3892/mmr.2019.10660 PubMed Abstract | 10.3892/mmr.2019.10660 | Google Scholar 31545489 PMC6797988

[B84] Wang H. Liu C. Zhao Y. Gao G. (2020). Mitochondria regulation in ferroptosis. Eur. J. Cell Biol. 99, 151058. 10.1016/j.ejcb.2019.151058 PubMed Abstract | 10.1016/j.ejcb.2019.151058 | Google Scholar 31810634

[B85] Watson J. (2013). Oxidants, antioxidants and the current incurability of metastatic cancers. Open Biol. 3, 120144. 10.1098/rsob.120144 PubMed Abstract | 10.1098/rsob.120144 | Google Scholar 23303309 PMC3603456

[B86] Wu Z. Geng Y. Lu X. Shi Y. Wu G. Zhang M. (2019). Chaperone-mediated autophagy is involved in the execution of ferroptosis. Proc. Natl. Acad. Sci. U. S. A. 116, 2996–3005. 10.1073/pnas.1819728116 PubMed Abstract | 10.1073/pnas.1819728116 | Google Scholar 30718432 PMC6386716

[B87] Xu S. (2019). Iron and atherosclerosis: The link revisited. Trends Mol. Med. 25, 659–661. 10.1016/j.molmed.2019.05.012 PubMed Abstract | 10.1016/j.molmed.2019.05.012 | Google Scholar 31230908

[B88] Yamada N. Karasawa T. Wakiya T. Sadatomo A. Ito H. Kamata R. (2020). Iron overload as a risk factor for hepatic ischemia-reperfusion injury in liver transplantation: Potential role of ferroptosis. Am. J. Transpl. 20, 1606–1618. 10.1111/ajt.15773 PubMed Abstract | 10.1111/ajt.15773 | Google Scholar 31909544

[B89] Yang M. Chen P. Liu J. Zhu S. Kroemer G. Klionsky D. J. (2019). Clockophagy is a novel selective autophagy process favoring ferroptosis. Sci. Adv. 5, eaaw2238. 10.1126/sciadv.aaw2238 PubMed Abstract | 10.1126/sciadv.aaw2238 | Google Scholar 31355331 PMC6656546

[B90] Yang H. Luo F. Wei Y. Jiao Y. Qian J. Chen S. (2021). TGR5 protects against cholestatic liver disease via suppressing the NF-κB pathway and activating the Nrf2/HO-1 pathway. Ann. Transl. Med. 9, 1158. 10.21037/atm-21-2631 PubMed Abstract | 10.21037/atm-21-2631 | Google Scholar 34430599 PMC8350648

[B91] Zhang Y. Shi J. Liu X. Feng L. Gong Z. Koppula P. (2018). BAP1 links metabolic regulation of ferroptosis to tumour suppression. Nat. Cell Biol. 20, 1181–1192. 10.1038/s41556-018-0178-0 PubMed Abstract | 10.1038/s41556-018-0178-0 | Google Scholar 30202049 PMC6170713

[B92] Zhang W. Sun Y. Bai L. Zhi L. Yang Y. Zhao Q. (2021). RBMS1 regulates lung cancer ferroptosis through translational control of SLC7A11. J. Clin. Invest. 131, e152067. 10.1172/jci152067 PubMed Abstract | 10.1172/jci152067 | Google Scholar 34609966 PMC8592553

[B93] Zhang Y. Kong Y. Ma Y. Ni S. Wikerholmen T. Xi K. (2021). Loss of COPZ1 induces NCOA4 mediated autophagy and ferroptosis in glioblastoma cell lines. Oncogene 40, 1425–1439. 10.1038/s41388-020-01622-3 PubMed Abstract | 10.1038/s41388-020-01622-3 | Google Scholar 33420375 PMC7906905

[B94] Zhou Y. Que K. T. Zhang Z. Yi Z. J. Zhao P. X. You Y. (2018). Iron overloaded polarizes macrophage to proinflammation phenotype through ROS/acetyl-p53 pathway. Cancer Med. 7, 4012–4022. 10.1002/cam4.1670 PubMed Abstract | 10.1002/cam4.1670 | Google Scholar 29989329 PMC6089144

[B95] Zhu S. Zhang Q. Sun X. Zeh H. J. 3rd Lotze M. T. Kang R. (2017). HSPA5 regulates ferroptotic cell death in cancer cells. Cancer Res. 77, 2064–2077. 10.1158/0008-5472.Can-16-1979 PubMed Abstract | 10.1158/0008-5472.Can-16-1979 | Google Scholar 28130223 PMC5392369

[B96] Zhu Y. Xian X. Wang Z. Bi Y. Chen Q. Han X. (2018). Research progress on the relationship between atherosclerosis and inflammation. Biomolecules 8, E80. 10.3390/biom8030080 PubMed Abstract | 10.3390/biom8030080 | Google Scholar 30142970 PMC6163673

[B97] Zucca F. A. Segura-Aguilar J. Ferrari E. Muñoz P. Paris I. Sulzer D. (2017). Interactions of iron, dopamine and neuromelanin pathways in brain aging and Parkinson's disease. Prog. Neurobiol. 155, 96–119. 10.1016/j.pneurobio.2015.09.012 PubMed Abstract | 10.1016/j.pneurobio.2015.09.012 | Google Scholar 26455458 PMC4826627

